# Periscope

**Published:** 1890-06

**Authors:** 


### PERISCOPE.

### MEDICINE.

**A Case of Erysipelatous Broncho-pneumonia without External Erysipelas.**—M. Brouardel reports this case of broncho-pneumonia, which, as will be seen below, was clearly of erysipelatous nature. A woman of thirty-seven, who had nursed her master, suffering from facial erysipelas, since December 22nd, was taken with rigors and a pain in the side on December 23rd, admitted December 24th with pneumonia at the base of the right lung, and died on the evening of December 25th. She showed no trace of erysipelas on the mucous or cutaneous surfaces. At the autopsy a very limited patch of broncho-pneumonia, with the usual anatomical characters, was found. Bacteriological examination of scrapings of the exudation, and of sections stained by Gram's method, demonstrated the strepto-coccus of erysipelas. Characteristic colonies of the strepto-coccus were obtained by cultures, unmixed with any other organism. Injection of three drops of a culture underneath the skin of a rabbit's ear produced a typical attack of erysipelas in the animal, from which it recovered with the formation of an abscess. M. Brouardel believes that is the first case in which the erysipelatous origin of a broncho-pneumonia has been conclusively demonstrated.—*Gazette des hôpitaux*, February 13th, 1890. J. M. C.

**Varieties of Angina Pectoris.**—M. Rendu reports the case of a man who suffered from attacks of angina pectoris and had signs of aortic valve disease, but in whom the symptoms did not indicate extensive damage to the valves. The symptoms, though they resembled, did not altogether correspond to, those of true angina; and in his remarks on the case M. Rendu says that he believes the group of symptoms characterising true angina may result from a simple neuralgia, from the effects of tobacco, of some poison, or even of dysmenorrhœa. He thinks that cases of angina pectoris may be placed in one of two groups: (1) those in which the angina is symptomatic of an organic lesion, *e.g.* atheroma of coronary arteries, or of a neuritis of the cardiac plexus; and (2) those in which anginal attacks form part of a neurosis, *e.g.* in Graves' disease. In the latter, the symptoms are more alarming than dangerous, and may be distinguished from attacks of genuine angina by the following characteristics:

(1) Attacks frequently nocturnal, and without exciting cause.

(2) Often preceded by an aura, of longer or shorter duration, from the periphery.

(3) Pain diffused over a wider area, with often intense cutaneous hyperæsthesia over præcordial area, resembling an hysterogenous zone.

(4) Accompanied by violent and not by lessened heart-beats, by sighing but not interrupted respiration, and sometimes by actual loss of consciousness.

(5) The attacks are more frequent than in the true form. The diagnosis between them may, however, be very difficult when aortic disease co-exists with the neuropathic state.—*Gazette des hôpitaux*, January 2nd, 1890. J. M. C.

**The Differential Diagnosis between Simple and Tubercular Broncho-pneumonia in Children.**—M. Hutinel attaches weight to the following considerations:

In tubercular broncho-pneumonia, onset is more insidious. Dyspnœa is excessive; that is to say, it is greater than the auscultatory and other physical signs of consolidation would lead us to expect. There are perhaps a few scattered râles to be heard, with a small patch of deficient resonance; and yet, with these slight signs, there may be 50—60 respirations to the minute, and marked cyanosis. Speaking generally, the auscultatory signs are more prominent in simple inflammatory broncho-pneumonia.

As to the localisation of the mischief, the apices are far less frequently affected in children than in adults. In children under the age of six to seven, it is rare to find tubercular consolidation limited to the apices. On the other hand, a simple broncho-pneumonia may affect the apex, and a tubercular the base only, of the lung. The temperature gives no certain indications; but in the tubercular variety temperature and pulse often do not correspond. The pulse may be 140—150 beats to the minute, and the temperature 100°; or the temperature may be high and the pulse slow.

Convulsions have little significance, unless there be in addition other symptoms of meningitis present. Evidence of presence of tubercle in other organs must be carefully sought for. Often the diagnosis can only be certainly made by watching the general course of the malady; in the tubercular cases, of course, normal resolution does not show itself: from time to time the auscultatory signs may indeed improve, but only to be succeeded by fresh mischief, while the general condition of the little patient deteriorates.—*Gazette des hôpitaux*, January 30th, 1890. J. M C.

**Morvan's Disease.**—A lecture of Professor Charcot's is devoted to the consideration of a disease to which Dr. Morvan, of Brittany, first drew attention in 1883. The disease affects the upper extremities, and the chief symptoms are: (1) neuralgic pains; (2) paresis, with analgesia, first on one side, then on both; (3) the appearance later on of whitlows. Sometimes paresis and analgesia come before the pains; there are often other trophic disturbances, and in some cases these, with the presence of whitlows, are the only symptoms, paresis and analgesia being absent. In typical cases there are, first, neuralgic pains; then paresis, with more or less muscular atrophy in the hand and forearm; and, finally, loss of sensation to touch, pain, and temperature, generally affecting the hand, forearm, and part of the arm, but sometimes spreading over the whole of the upper extremity. The whitlows appear later. There is always necrosis of the bones, most commonly of the last phalanx, and characteristic deformations are thus produced. There may be as many as seven, eight, or nine whitlows, scattered over a long period of time (twelve to forty years). Generally there is no pain with these whitlows; but those that appear first may be painful, those that come later never are. Other trophic disturbances are, deep fissures of the skin, and a sort of perforating ulcer of the hand, which may open up the sheaths of the tendons and cause suppuration in them. The nails fall off or become much deformed. The hand becomes livid, its temperature is sub-normal, and it may be subject to excessive sweating. The lower extremities are very rarely affected; there is often scoliosis of the vertebral column.

The duration of the disease is long—ten, fifteen, twenty years, or more. The etiology is unknown: so far, men seem to be affected more often than women; it may come on at any age.

The maladies from which the diagnosis is most difficult are, scleroderma, anæsthetic leprosy, and syringomyelia.—*Le Progrès médical*, March 15th, 1890. J. M. C.

**The Difference between "Specific" and "Non-specific" Diseases.**—Professor Bouchard reviews the theories formerly held on this subject, and shows that they do not meet the facts of the case. It has been held in recent years that contagiousness and the power of conferring immunity from fresh attacks are the two most distinctive properties of specific diseases. But although this is true of the extreme cases, it will not hold good for all the variations and degrees of severity of so-called specific diseases. As shown by culture and inoculation-experiments, the property of contagiousness may spontaneously diminish or even disappear, whilst under certain conditions non-specific diseases become contagious.

Professor Bouchard considers that the only difference between specific and non-specific diseases lies in the different character of the microbes to which they are due: those which produce the latter diseases are constantly in us or upon us, as a sort of parasite; but under ordinary conditions we are completely protected from them. When, however, they gain, by some means, admittance into the blood-circulation, they produce morbid reaction. There is, for instance, a diplococcus generally present in the buccal secretions of man which is fatal to mice in twenty-four hours. On the other hand, in the specific diseases the germs are never found about us under normal conditions: in these the presence of the microbe is everything, and the condition of the soil on which it grows is of minor importance; the organism, even when in good health, is without defence against these germs. In the non-specific diseases, there is nothing to fear from the microbes if the organism is in healthy condition; they cannot then produce their morbific effects.—*L' Union médicale*, April 17th, 1890. J. M. C.

**Centripetal Venous Pulse.**—This pulse, which receives also the name of "ascending venous pulse," is that observed in the superficial veins of the arms, and is but very seldom seen. Quincke, however, does not believe it to be so rare as represented, and states that he has seen it many times. The principal condition necessary for its appearance is relaxation of the blood-vessels, more especially of the arteries; but it requires also dilatation of capillaries and veins, as that which accompanies certain stages of fevers where there are sudden crises with sweating. Thus he has seen it in typhoid, relapsing, intermittent, pyæmia, polyarthritis rheumatica, pneumonia, phthisis and gall-stones. Nervous influence might be present along with fever in cases of meningitis, spondylitis, encephalo-malacia and cervical injuries. It may be observed, likewise, in apyretic states, chlorosis, traumatic anæmia and cancer, as well as in the healthy if the summer-heat has induced relaxation of the peripheral vessels. The phenomenon is observed only on the back of the hand and forearm; but once only did Quincke see it in the veins of the dorsum of the foot. Besides the above, other conditions are essential—softness of the skin, powerful heart's action producing a sufficiently high pulse-wave and a certain medium degree of filling of the veins: the pulse easily disappears, mere change of position being sufficient to efface it. The presence of a centripetal venous pulse does not necessarily indicate that a capillary pulse must also be visible: perhaps a direct continuity of artery and vein may explain this. The transmission of the pulse-wave into the veins is the more likely, the greater the pressure-difference between the cardiac systole and diastole. In the extreme arterial relaxation of the later stages of fever this is most marked, as also in aortic regurgitation. It may be favoured, moreover, by arterio-sclerosis (Senator), and retarded by venous stagnation (Holz). For the occurrence of the visible capillary pulse and the centripetal venous pulse we have, in common, the height and rapidity of the pulse-wave, and, in addition, there is necessary for the latter extensive relaxation of the large arteries and veins.—*Berliner klinische Wochenschrift*, No. 12, 1890. D. R. P.

**The so-called "Nona."**—Dr. Tranjen, of Sistow (Bulgaria), records two cases observed during the month of April, the symptoms of which suggested the condition of which much has been heard of in the lay papers under the name of "Nona." The first was a child two years old, of healthy family, seen in the third week of illness. It appeared to be asleep. It moaned now and then, and cried and grasped its head; and was said to have been in that state for three weeks. It could be readily awakened and induced to take bread-and-milk: often it awoke of its own accord and called for water. The bowels were constipated, and had no motion without castor oil. For urination the child woke up. The little patient was well-formed; and lay on its side, with the head thrown back. The large fontanelle was closed; the pupils equal, dilated, and reacting slowly to light; the tongue coated; and the abdomen retracted. There was no rash, no splenic enlargement, and no discoverable organic disease. The temperature did not rise above 101°, and the pulse and respiration were apparently normal. This condition lasted sixteen days; then coma set in, and death two days later.

The other instance was that of a ten-year-old anæmic and emaciated girl. Her parents were healthy. She died, with symptoms similar to the other, after seven days' illness. In January she had influenza for three weeks. In

April she fell into a sleepy state, with fever and constipation, retracted abdomen, coated tongue, head drawn back, and pupils unequal, dilated, and reacting slowly. She awoke occasionally and drank something, embraced her mother, and then fell asleep again. She did not speak nor ask for anything. Shortly before death, slight trismus set in. The temperature was no higher than 100.5°, and the pulse and respiration not abnormal.

These two cases reminded the writer of a soldier who died, after ten days' illness, with the same symptoms, during the influenza epidemic in January. The *post mortem* examination showed well-marked hyperæmia of the meninges, an œdematous condition of the brain, but no exudation.

Tranjen looks upon these cases as infectious cerebro-spinal meningitis. The signs of irritation were very few, and the prominence of the drowsiness would impress a lay mind very much. The sleep, abnormal in its duration for days and weeks, was otherwise physiological. Clinically and pathologically, the condition was none other than a cerebro-spinal meningitis running an unusual course, and resembling pneumonia very much in its relation to influenza.—*Berliner klinische Wochenschrift*, No. 22, 1890. D. R. P.

### THERAPEUTICS.

**Alkaloids of Cod-liver Oil.**—Mm. Gautier et Morgues. The beneficial action of cod-liver oil is due to three principal factors:

(1) Fats, which are easily assimilated, on account of their partial saponification, the small amount of fatty acids present, and the presence of bile salts, which render their emulsification extremely easy, even without the aid of the digestive ferments. If cod-liver oil is shaken up with water containing a trace of alkali, or of an alkaline carbonate, a very fine emulsion is formed. To these fats a large share of the efficacy of the oil is due.

(2) The oil is rich in phosphates, phosphoric acid, lecithin and phosphorus in organic combination; the last form is the most readily assimilated. There is also a small quantity of iodine and bromine.

(3) Alkaloids, of which there are a great number; butylamine, amylamine, morrhuine and morrhuic acid. These are nerve stimulants, render more active the nutritive processes, increase the amount of urine and sweat, and at the same time promote appetite. Some among these, notably amylamine, are, in large doses, powerful poisons: the latter, in smaller doses, increases reflex action and produces convulsive tremblings; in the feeble doses in ordinary use, they merely stimulate the nutritive functions.—*Gazette des hôpitaux*, February 6th, 1890. J. M. C.

**Chloralamide.**—M. Bilhaut, in a paper on this hypnotic, concludes that chloralamide is an efficient hypnotic; and, since it has no injurious action on the heart or kidneys, may be used in patients who suffer from heart-disease or albuminuria. It does not lose its effect when given every other night regularly for ten weeks. Its narcotising properties are not destroyed when it is dissolved in boiling water. The dose is from 30—75 grains, given at bedtime in one dose.—*Le Progrès médical*, February 1st, 1890. J. M. C.

**Orexin.**—Prof. Penzoldt has recently introduced, as a stomachic tonic, a substance to which he has given the name of orexin. It is one of the phenol derivatives, the chloride of the substance being readily soluble. In experiments, an early sensation of hunger was produced, and this action was found useful in the diminished appetite of phthisis and in anœrexia after severe operations. The drug was given in the form of gelatinised pill, in doses of 5 to 8 grains daily. Glückziegel has tested it in 17 patients suffering from various affections. In the majority of those the result was satisfactory; in three there was no effect on the appetite, vomiting occurring in one case, and pain across the stomach in another. On the other hand, Imrédy had not so much success. The drug was not well borne, produced an inclination to vomit, and increased the appetite only in seven out of eleven cases, the other four remaining without any action.—*Therapeutische Monatshefte*, Nos. 2 and 5, 1890. D. R. P.

**Pyo-etanin.**—This name has been given by Merck to a new preparation from the aniline dyes. He has introduced it as a disinfectant and dressing, suggested by the observations of Stilling and Wortmann. Stilling has found that methyl violet, free from arsenic, restrains the growth of germ cultures in a solution of 1 to 30,000, and destroys them at a strength of 1 to 10,000. Injurious effects were not seen in trials on animals, and the practical results, which were obtained first of all in some eye diseases and suppurating wounds of different kinds, were very satisfactory. The drug is used in the form of a powder dusted over the parts, and as readily-soluble pastilles, pencils and ointments; and dressings are impregnated with it. The very intense colour, which is a drawback, can be easily destroyed by spirit or mineral acids. —*Berliner klinische Wochenschrift*, No. 22, 1890. D. R. P.

